# Management of Fractured Fragment With Reattachment: A Case Report With 2‐Year Follow‐Up

**DOI:** 10.1155/crid/2224243

**Published:** 2026-10-17

**Authors:** Manigandan Kuzhanchinathan, Tharani Jayarajapandian, Tharun Kumar Sankar, Mathan Rajan Rajendran

**Affiliations:** ^1^ Department of Conservative Dentistry and Endodontics Sri Ramachandra Dental College and Hospital Sri Ramachandra Institute of Higher Education and Research (Deemed to Be University) Chennai Tamil Nadu India

**Keywords:** fragment reattachment, traumatic dental injuries, uncomplicated crown fracture

## Abstract

Fragment reattachment is the most conservative treatment approach for uncomplicated crown fractures, which enables the restoration of the original tooth structure and the preservation of the remaining tooth structure. A 15‐year‐old patient reported with an uncomplicated crown fracture of the maxillary left central incisor involving enamel and dentin. Pulp sensibility testing had a normal response and radiographic examination revealed no periapical pathology. The retrieved fractured fragment had good marginal adaptation. Following rehydration, fragment reattachment was performed under rubber dam isolation using an etch‐and‐rinse protocol, a two‐coat adhesive application and nanohybrid flowable composite. At the second appointment, a V‐shaped retentive groove of 0.7‐mm depth was placed in both the labial and palatal aspects, extending 2 mm on either side of the fracture line, followed by composite restoration. The patient was reviewed at 6 weeks, 1 year and 2 years in accordance with the International Association of Dental Traumatology (IADT) guidelines. Throughout the follow‐up period, the reattached fragment remained clinically intact with no evidence of debonding, marginal discolouration or fracture recurrence. Pulp sensibility testing remained normal, and radiographic examination revealed no periapical changes at all follow‐up intervals, demonstrating sustained aesthetic and functional stability over a 2‐year follow‐up period.

## 1. Introduction

Traumatic dental injuries (TDI) represent the most prevalent dental emergencies, with maxillary anterior teeth accounting for approximately 33% of such injuries [1, 2]. Among these, maxillary central incisors are predominantly affected comprising 47% due to their prominent position in the dental arch [1]. The aetiology of TDI includes road traffic accidents, sports‐related injuries, falls and assault [3]. Beyond the compromise in both aesthetics and function, these injuries exert a significant impact on the social and psychological well‐being of the patient [4]. Additionally, these can also adversely affect speech and mastication [5].

Several classifications have been proposed in the literature with respect to TDI [6–10]. The latest International Association of Dental Traumatology (IADT) guidelines recommends descriptive terminology for crown fractures. Accordingly, injuries involving enamel and dentin without pulpal exposure are currently classified as uncomplicated crown fractures [11]. The extent of trauma to the tooth and the supporting structures primarily depends on the amount of energy delivered during frontal impact to the anterior teeth region. When the impact energy is greatly absorbed by the crown and limits transmission to the root and the supporting structures, it results in crown fracture without displacement [8].

Uncomplicated crown fracture denotes fracture of the crown involving enamel and dentin with no pulpal exposure [7], which has the highest prevalence rate of 19% [8]. When left untreated, these fractures can result in dentin hypersensitivity, aesthetic compromise and functional impairment [12]. In such cases, restoration of both the aesthetics and function remains the primary aim of the treatment.

Conventional treatment methods include restoration with direct composite layering techniques [13] or indirect restoration approaches, which may require additional modification of the residual tooth structure [14]. When the fractured fragment is available and intact, reattachment serves as the most conservative and aesthetic treatment approach that enables preservation of the remaining tooth structure while restoring the original tooth morphology [11]. Fragment reattachment was first reported in the literature by Chosack and Eidelmanin [15] and has since exhibited a success rate of about 82.8% [16]. The success rate mainly depends on the adaptation of the fractured fragment to the tooth surface, the extraoral dry time and the orientation of the fracture plane. Based on the orientation of the fracture line, crown fractures have been described in three types. Type A, in which the fracture plane is angled cervically in a lingual to facial direction when viewed in the proximal aspect; Type B, in which the fracture plane is angled cervically in a facial to lingual direction when viewed in the proximal aspect; and Type C, in which the fracture line is approximately perpendicular to the long axis of the tooth. Among these, Type C fractures are considered to have comparatively poor retention following fragment reattachment, owing to the unfavourable fracture geometry and reduced surface area available for bonding. Therefore, reattachment of fragments associated with Type C fracture orientation may present a greater biomechanical challenge and require careful case selection and an appropriate adhesive protocol.

Although fragment reattachment is an established procedure, the majority of published case reports primarily focused on the immediate postoperative outcome. Case reports with follow‐up period beyond 1 year remain limited. Furthermore, the influence of fracture plane orientation on the biomechanical retention of the reattached fragment and the role of the adhesive strategies have received limited clinical discussion.

The present case report addresses both these gaps by documenting the management of an unfavourable crown fracture with a Type C fracture orientation—described as the horizontal fracture line perpendicular to the long axis of the tooth usually associated with comparatively reduced retention—which was managed to maintain sustained clinical and radiographic stability over a 2‐year follow‐up period, thereby contributing to the previous evidence for this treatment modality.

This case report was prepared in accordance with the CARE guidelines for case reports [17]. An informed consent was obtained from the patient′s parent as per the recommendations of the International Committee of Medical Journal Editors (ICMJE) to publish this case report.

## 2. Case Presentation

### 2.1. Chief Complaint

A 15‐year‐old patient reported to the outpatient department (OPD) of Conservative Dentistry and Endodontics, Sri Ramachandra Dental College and Hospital with a chief complaint of broken tooth in the upper front teeth region.

### 2.2. History of Trauma

The patient gave a history of trauma to the maxillary left central incisor while playing on the school grounds 1 day prior to reporting to OPD.

### 2.3. Extraoral Examination

No history of epistaxis, loss of consciousness or soft tissue laceration in and around the oral cavity was evident during or following the trauma. No evidence of extraoral swelling or signs of trauma on the day of examination. Patient was systemically healthy.

### 2.4. Clinical Examination

During intraoral examination, an oblique fracture was evident in relation to 21 extending from the cervical one third of the mesial aspect to the incisal third of the distal aspect in 21 involving enamel and dentin with no clinical pulpal exposure suggestive of uncomplicated crown fracture (Figure 1). No mobility of the injured tooth was observed. The fractured segment was kept in a water‐filled container and was brought by the patient (Figure 2).

**Figure 1:**
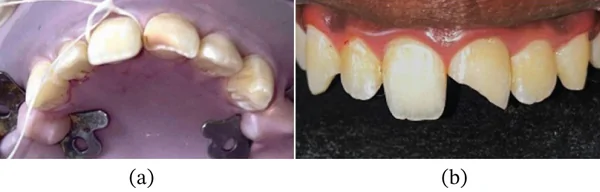
Preoperative image: (a) occlusal view and (b) frontal view.

**Figure 2:**
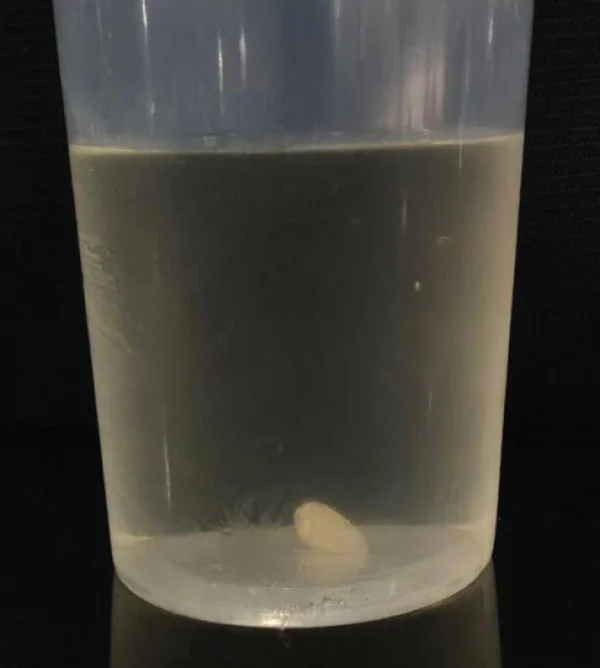
Fractured fragment stored in a water‐filled container and brought by the patient.

### 2.5. Pulp Sensibility Testing

Pulp sensibility testing was performed on both test and control teeth using Endo‐Frost (Coltène/Whaledent GmbH+ Co. KG, Langenau, Germany) sprayed on a cotton pellet (size 2). Placement of cotton pellet on the midlabial surface of the control Tooth 11 elicited immediate response upon application that returned to normal upon removal of the stimulus. Similar response was observed with respect to the test Tooth 21. In addition, electric pulp testing was performed using an electric pulp tester (Parkell, Inc., 300, New York 11717, United States). After isolation, the electrode tip was coated with toothpaste, which acts as a conducting medium. The tip was positioned on the surface of the tooth to be examined and the lip clip was positioned to complete the circuit. The value at which the patient experienced the tingling sensation was recorded on the pulp tester and compared with the test tooth. The readings obtained were around ‘5’, which is similar to both the control and the test teeth.

### 2.6. Radiographic Examination

Radiovisiography (RVG) in relation to 21 revealed radiolucency involving enamel and dentin suggestive of crown fracture with respect to 21. No periapical change was evident in 21 (Figure 3).

**Figure 3:**
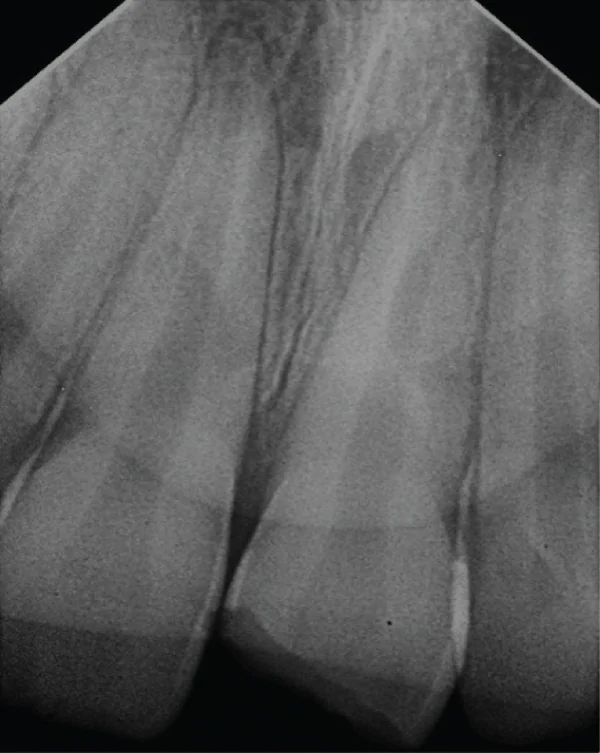
Preoperative radiograph showing radiolucency involving enamel and dentin in relation to 21 with no periapical changes.

### 2.7. Assessment of the Fractured Fragment

The fractured fragment was assessed to evaluate the condition of the fragment, rule out the presence of secondary fracture lines and to ensure complete availability of the fractured portion.

### 2.8. Diagnosis

Based on the clinical findings, pulp sensibility testing and radiographic findings, Tooth 21 was diagnosed as reversible pulpitis (associated with uncomplicated crown fracture) with normal periapical tissues.

### 2.9. Treatment

Considering the age of the patient, the line of fracture, occlusion, clinical and radiographic findings, reattachment of the fractured fragment was considered as the treatment option.

## 3. Procedure

### 3.1. Informed Consent

All the information regarding the treatment procedure, number of visits, outcome expected and the prognosis were clearly explained to the patient′s parent (as the patient was 15 years old). Following which, informed consent was obtained from the patient′s parent, encompassing all the details of the treatment procedure.

### 3.2. Fragment Rehydration

The fractured fragment was immersed in saline for around 30 min to facilitate rehydration prior to bonding [18] (Figure 4).

**Figure 4:**
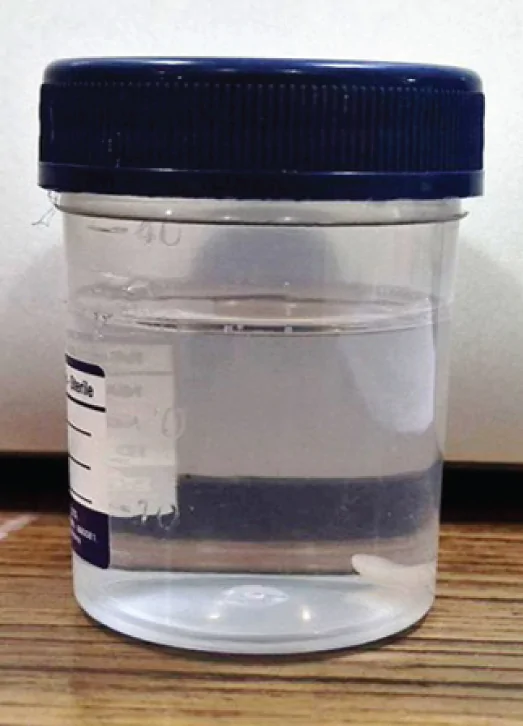
Fragment immersed in saline medium for rehydration.

### 3.3. Rubber Dam Isolation and Trial Approximation

Rubber dam was used to isolate the operating field and to ensure adequate moisture control (Figure 5). The fractured fragment was then approximated in position without bonding to evaluate the marginal adaptation and the final aesthetic outcome.

**Figure 5:**
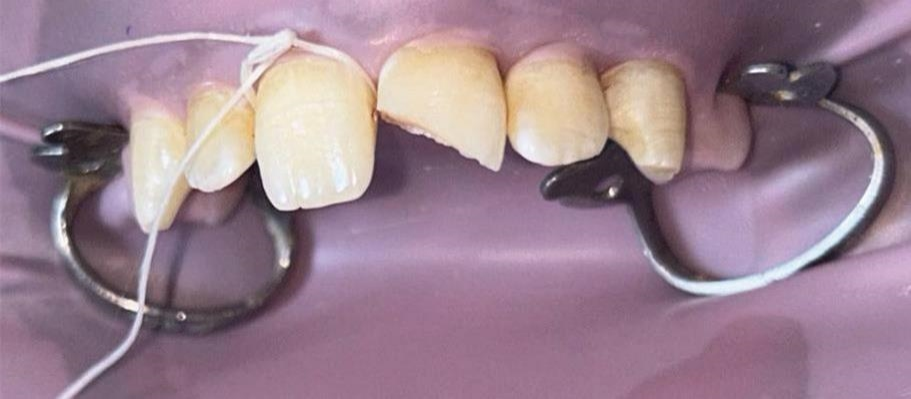
Rubber dam isolation.

### 3.4. Etching Protocol

The clinical crown of central incisor and the fractured fragment were etched with 37% orthophosphoric acid for 15 s (Etching Liquid, Prime Dental Products Private Ltd., Maharashtra, India) (Figure 6). The etchant was thoroughly rinsed off and blot dried.

**Figure 6:**
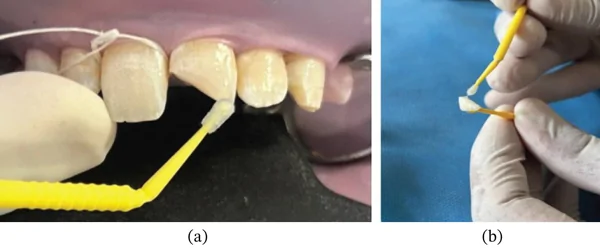
Etching with 37% orthophosphoric acid: (a) clinical crown and (b) fragment.

### 3.5. Bonding Protocol

A two‐coat application of adhesive (Meta‐P&B, Meta Biomed Co., Cheongju, Korea) was done on the substrate of the clinical crown and the fractured fragment with disposable micro brush. The adhesive was air dried gently to evaporate the solvent (Figure 7).

**Figure 7:**
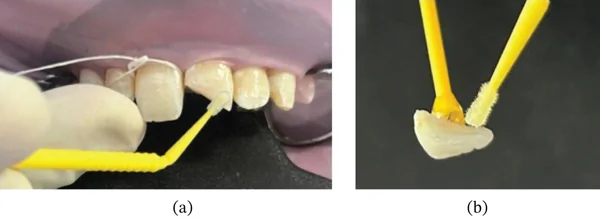
Application of the adhesive: (a) clinical crown and (b) fragment.

### 3.6. Fragment Reattachment

Nanohybrid flowable composite (Nexcomp Flow, Meta Biomed Co., Cheongju, Korea) was applied as a thin layer to both clinical crown and the fractured fragment and was reapproximated. Tack curing was done for 5 s using a light curing unit (Woodpecker LED D, Guilin Woodpecker Medical Instrument Co. Ltd., Guilin, China), which delivers light intensity/irradiance ranging from 1000 to 1700 mW/cm^2^ operating at a wavelength of 420–480 nm. Excess composite was removed using the Bard Parker blade (No. 15) and later light cured from both buccal and palatal sides for 40 s (Figure 8).

**Figure 8:**
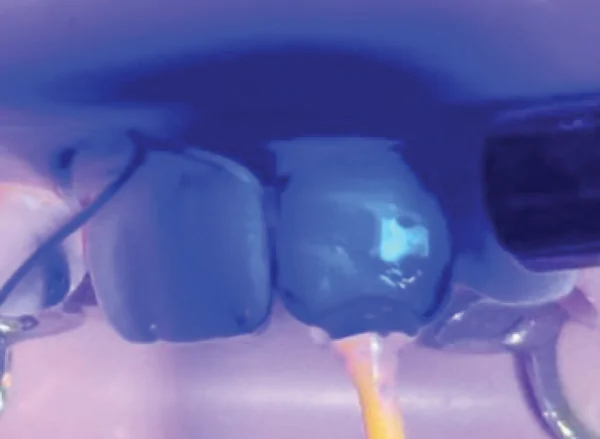
Approximation of the fractured fragment to the clinical crown with flowable composite and light cured.

### 3.7. Rubber Dam Removal and Occlusal Adjustment

Following light curing, the rubber dam was carefully removed by cutting the interseptal portion of the dam, and the dam sheet was withdrawn. Occlusion was carefully evaluated in protrusive and lateral movements and adjusted to eliminate any premature contacts. Postoperative radiograph was taken to confirm the approximation of the fractured segment to the clinical crown (Figure 9). Instructions regarding dietary precautions, oral hygiene maintenance and the care with respect to the treated tooth were given to the patient. The patient was kept under observation and follow‐up.

**Figure 9:**
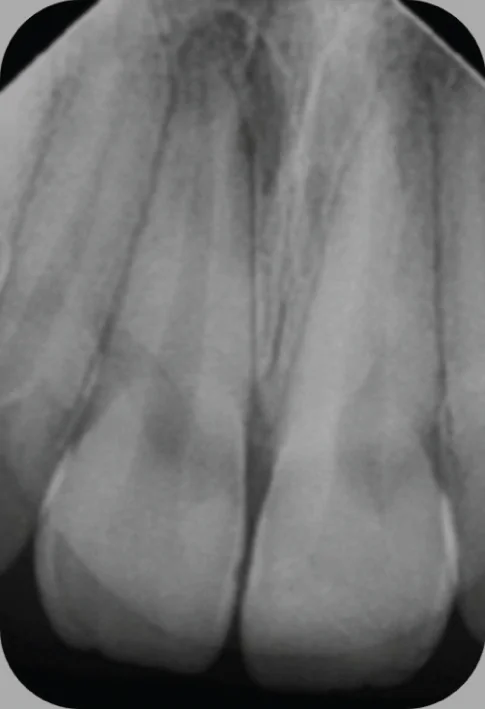
Postoperative radiograph showing the approximated fragment to the clinical crown.

### 3.8. V‐Shaped Internal Groove

After 3 days, in the second appointment, rubber dam isolation was re‐established. In order to improve the retention and the transition line, a V‐shaped groove with a depth of 0.7 mm was given in both labial and palatal aspect extending 2 mm on either side of the fracture line (Figure 10).

**Figure 10:**
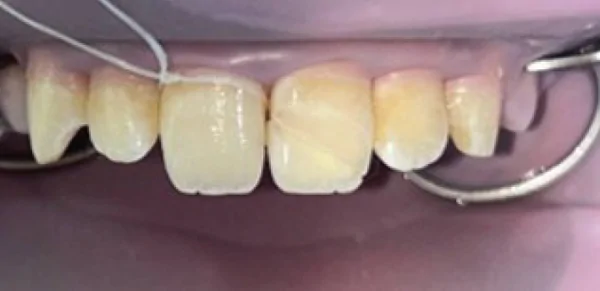
Placement of the retentive grooves.

### 3.9. Shade Selection and Composite Restoration

In order to select the main dentinal shade, a minimal amount (‘button‐try’) of different shades of composite closely matching the original shade was placed over the clinical crown portion of the central incisor and light cured. Photographs were taken and converted to monochrome to match the value of the tooth to be restored with the composite resin. The shade that blends with the shade of the tooth was selected. The prepared clinical crown portion was etched with 37% orthophosphoric acid for 15 s, rinsed and blot dried. A two‐coat application of adhesive was applied with disposable micro brush and air dried gently to evaporate the solvent and is light cured. Restoration of the groove was done with the selected composite resin (G‐aenial composite resin [OA2], GC Corporation, Tokyo, Japan).

### 3.10. Finishing and Polishing

Initial finishing and contouring of the composite resin was done with carbide bur. Final polishing was performed in multiple steps using the abrasive discs (Sof‐Lex, 3M ESPE, St. Paul, Minnesota, United States) and silicone points (Enhance, Dentsply Sirona, York, Pennsylvania, United States). The final gloss and surface smoothness were obtained with diamond polishing paste (Prisma Gloss, Dentsply Sirona, York, Pennsylvania, United States) (Figure 11).

**Figure 11:**
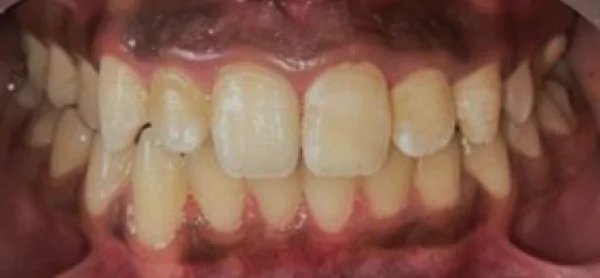
Immediate postoperative image after finishing and polishing.

### 3.11. Follow‐Up Period

From the time of clinical presentation, the patient underwent fragment reattachment in two appointments, which was completed within 4 days following the traumatic injury. Following treatment, patient was followed up according to the IADT guidelines at 6 weeks, 1 year and 2 years [11]. At each follow‐up period, radiographs were taken, and pulp sensibility testing was done to verify the pulpal and periapical status of the tooth. The attached fragment was checked clinically. Radiographic examination at every follow‐up period revealed no periapical changes. Pulp sensibility was evaluated in both the control and the test teeth with the thermal and electric testing methods, which revealed normal response. Throughout the follow‐up period, no fractures or marginal discolorations were evident. The aesthetic and functional stability was preserved (Figures 12, 13, 14 and 15).

**Figure 12:**
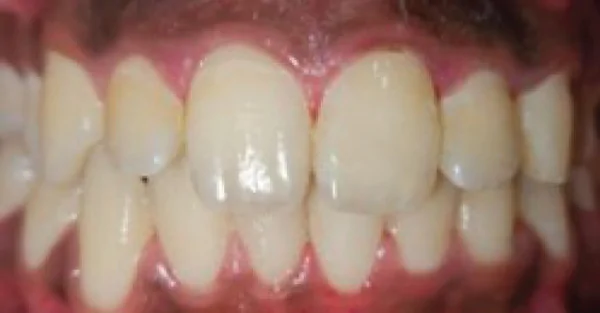
1‐year follow‐up image.

**Figure 13:**
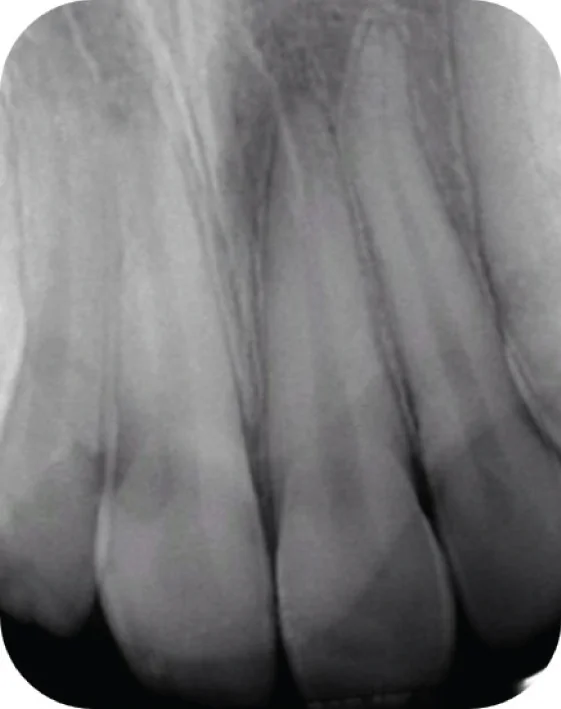
Radiograph at 1 year.

**Figure 14:**
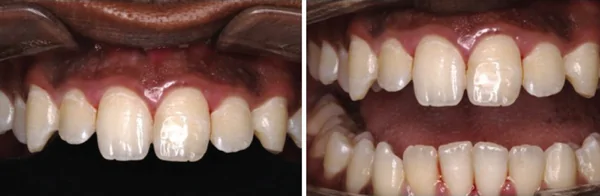
2‐year follow‐up images.

**Figure 15:**
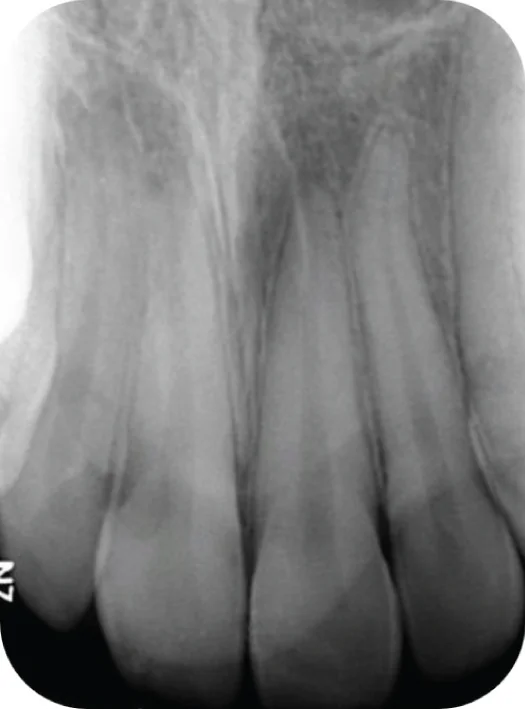
Radiograph at 2 years showing approximated fragment with no periapical changes.

## 4. Discussion

The present case report highlights the importance of thorough pretreatment clinical and radiographic assessment of the tooth along with strict adhesive protocols in achieving a predictable outcome in fragment reattachment. The main findings include the excellent marginal adaptation, normal pulp sensibility at presentation and at the follow‐up periods, better aesthetic and functional stability over a 2‐year follow‐up period. The incorporation of etch‐and‐rinse adhesive system, fragment rehydration and V‐shaped retentive groove preparation demonstrated successful reattachment of the fractured fragment in the maxillary left central incisor.

Crown fractures that were left untreated in the anterior region can result in dentinal hypersensitivity, poor aesthetics and function [12]. Advances in adhesive dentistry have made fragment reattachment a predictable and conservative treatment approach for such injuries [19]. The advantage of this approach is the ability to replicate the natural colour, translucency, optical properties, surface texture and morphology of the original tooth, which are clinically challenging through conventional direct composite restoration, and it requires considerable clinical skill and precision [20]. Additional clinical advantages include minimal tooth preparation and reduced chair‐side time [21].

Thorough pretreatment assessment is essential to determine the success of fragment reattachment. Evaluation of the fracture type, occlusal conditions and periodontal status of the affected tooth is important before the treatment plan [22]. The orientation of the fracture line is crucial as it has direct influence on restorability and long‐term prognosis [23]. In the present case, the oblique fracture represents a Type C orientation, featuring a fracture line perpendicular to the long axis of the tooth, which provides comparatively less mechanical support for the reattached fragment [23]. This highlights the importance of the adhesive protocol and retentive groove preparation to compensate for the reduced fracture strength of the tooth.

Based on the case understanding, a series of clinical steps were employed as follows: (1) clinical examination to determine the fracture extent, pulpal and periodontal status; (2) pulp sensibility testing using both thermal and electric pulp tests; (3) radiographic evaluation to rule out the periapical pathology; and (4) assessment of the fractured fragment for structural integrity, absence of secondary fracture lines and marginal adaptation. In the present case, all parameters were favourable as the fracture was oblique without pulp or root involvement, no occlusal interference, pulp sensibility was normal and the fragment demonstrated excellent marginal adaptation on trial approximation.

The hydration status of the fractured fragment is critical in achieving successful reattachment. Rehydration prior to bonding improves fracture resistance and facilitates recovery of optical properties between the fragment and the residual clinical crown [18, 21]. In the present case, the fragment was immersed in physiological saline for 30 min immediately prior to the bonding procedure. The duration of fragment rehydration was selected based on the previous evidence stating that dehydrated enamel and dentin could undergo dimensional changes, which would compromise marginal adaptation and affect optical coherence [18]. The 30‐min immersion period adopted in the present case was consistent with the previous literature, which demonstrated that rehydration periods of 20–30 min in saline were sufficient to achieve dimensional recovery of the dehydrated fragment the reattachment interface beyond, which no additional benefit was demonstrated [24].

The choice of adhesive system influences the durability of reattachment. The etch‐and‐rinse adhesive system was selected in preference to self‐etch alternatives on the basis of its well‐established superiority in enamel bonding. Phosphoric acid creates deep microporosities within the prism structure, which enables deeper resin tag penetration and hybrid layer [25]. Since this case predominantly involved enamel margins on both the residual tooth and the fragment, optimising enamel bond strength was most reliable and the primary consideration. Nanohybrid flowable composite resin was selected because of its low viscosity, high wettability, controlled application in thin, uniform increments, prevents fragment malpositioning and conforms greater fracture resistance [26]. To further enhance the retention and minimise the visibility of the fracture line, a V‐shaped internal groove was placed and subsequently restored with composite resin. This circumferential groove technique is more effective than simple direct bonding alone, as bevelled preparation orients enamel prisms perpendicularly, which increases the adhesive surface area and enables greater bond strength at the interface [27, 28]. Postirradiation polymerisation of the composite resins is a well‐documented phenomenon [29]. Polymerisation of composite resin could continue even after 24 h following irradiation, which could also increase the Knoop hardness as the residual free radicals continued the monomer conversion even in the absence of light [29, 30]. Hence, groove preparation was performed at a subsequent visit based on a clinical decision to allow the initially reattached fragment to remain undisturbed during the postpolymerisation period before performing additional preparation.

Long‐term clinical and radiographic follow‐up is important for the evaluation of traumatised teeth. When the pulpal complications arrive, they typically manifest within the first 6 months following injury; however, pulp necrosis may develop at later intervals, which underscores the importance of follow‐up [31]. In the present case, the patient was reviewed at 6 weeks, 1 year and 2 years in accordance with IADT guidelines [11]. At each interval, radiographic examination and pulp sensibility testing remained normal. No debonding, fragment fracture or marginal discolouration was observed at any follow‐up visit.

The limitation of this report is its single‐case design. As a single case report, it is only observational and does not attribute to outcome evaluation with the factors including fragment rehydration, selection of adhesive system or retentive groove preparation. The absence of comparator group such as direct composite or indirect restoration could have limited the advantages of the fragment reattachment procedure. Controlled clinical studies with larger sample sizes along with standardised protocols are required to establish the long‐term success of fragment reattachment.

The present case offers several practical key points in managing uncomplicated crown fractures with a retrieved fragment. A distinguishing feature of this case is the Type C fracture orientation, which is considered the least favourable type in the retention rates. This suggests that even in such types of fractures, careful attention to the adhesive protocol and the incorporation of retentive groove could serve as the key compensatory measures to provide favourable outcome with long‐term durability.

## 5. Conclusion

In the present case, management of uncomplicated crown fractures with fragment reattachment was associated with favourable aesthetic and functional outcomes over a 2‐year follow‐up period. The observations from the present case report suggest that fragment reattachment may represent a conservative and biologically sound treatment approach for managing uncomplicated crown fractures.

## Author Contributions

M.K. and T.K.S. were responsible for treatment planning, clinical case management and execution of the restorative procedure. T.J. was responsible for manuscript preparation, radiographic follow‐up and clinical documentation. M.K. and M.R.R. critically reviewed and evaluated the manuscript.

## Funding

No funding was received for this manuscript.

## Disclosure

All authors have read and approved the final version of the manuscript.

## Conflicts of Interest

The authors declare no conflicts of interest.

## Supporting information


**Supporting Information** Additional supporting information can be found online in the Supporting Information section. 

## Data Availability

The data that support the findings of this study are available from the corresponding author upon reasonable request.
