# Supplementary material for: Management of Fractured Fragment With Reattachment: A Case Report With 2‐Year Follow‐Up
Source: Case Rep Dent. 2026 Oct 17;2026:2224243. doi: 10.1155/crid/2224243 (PMC13675939; doi:10.1155/crid/2224243)
Supplement: Supplementary file 1 [file CRID-2026-2224243-s001.docx]

**CARE Checklist of information to include when writing a case report**

| **Topic** | **Item** | **Checklist item description** | **Reported on Line** |
| --- | --- | --- | --- |
| **Title** | **1** | The diagnosis or intervention of primary focus followed by the words "case report" | 1 (Page 1) |
| **Key Words** | **2** | 2 to 5 key words that identify diagnoses or interventions in this case report, including "case report" | 42-43 (Page 2) |
| **Abstract (no references)** | **3a** | Introduction: What is unique about this case and what does it add to the scientific literature? | 38-39 (Page 2) |
|  | **3b** | Main symptoms and/or important clinical findings | 26-28 (Page 2) |
|  | **3c** | The main diagnoses, therapeutic interventions, and outcomes | 26 (Page 2) |
|  | **3d** | Conclusion — What is the main "take-away" lesson(s) from this case? | 38-39 (Page 2) |
| **Introduction** | **4** | One or two paragraphs summarizing why this case is unique (may include references) | 80-85 (Page 4) |
| **Patient Information** | **5a** | De-identified patient specific information | 111-113 (Page 5) |
|  | **5b** | Primary concerns and symptoms of the patient | 113-116 (Page 5) |
|  | **5c** | Medical, family, and psycho-social history including relevant genetic information | 122-124 (Page 6) |
|  | **5d** | Relevant past interventions with outcomes | 123-124 (Page 6) |
| **Clinical Findings** | **6** | Describe significant physical examination (PE) and important clinical findings | 101-106 (Page 5) |
| **Timeline** | **7** | Historical and current information from this episode of care organized as a timeline | 194 – 197 (Page 8) |
| **Diagnostic Assessment** | **8a** | Diagnostic testing (such as PE, laboratory testing, imaging, surveys) | 107-126 (Page 8-9) |
|  | **8b** | Diagnostic challenges (such as access to testing, financial, or cultural) | - |
|  | **8c** | Diagnosis (including other diagnoses considered) | - |
|  | **8d** | Prognosis (such as staging in oncology) where applicable | - |
| **Therapeutic Intervention** | **9a** | Types of therapeutic intervention (such as pharmacologic, surgical, preventive, self-care) | - |
|  | **9b** | Administration of therapeutic intervention (such as dosage, strength, duration) | - |
|  | **9c** | Changes in therapeutic intervention (with rationale) | - |
| **Follow-up and Outcomes** | **10a** | Clinician and patient-assessed outcomes (if available) | - |
|  | **10b** | Important follow-up diagnostic and other test results | 193-203 (Page 9) |
|  | **10c** | Intervention adherence and tolerability (How was this assessed?) | - |
|  | **10d** | Adverse and unanticipated events | - |
| **Discussion** | **11a** | A scientific discussion of the strengths AND limitations associated with this case report | 268-285 (Page 12) |
|  | **11b** | Discussion of the relevant medical literature with references | - |
|  | **11c** | The scientific rationale for any conclusions (including assessment of possible causes) | - |
|  | **11d** | The primary "take-away" lessons of this case report (without references) in a one paragraph conclusion | 286-291 (Page 12) |
| **Patient Perspective** | **12** | The patient should share their perspective in one to two paragraphs on the treatment(s) they received | - |
| **Informed Consent** | **13** | Did the patient give informed consent? Please provide if requested **Yes ☐ No ☐** | Yes |
